# Fostering Reflection and Empathy: The Impact of Book Discussions on Medical Students' Education

**DOI:** 10.7759/cureus.94433

**Published:** 2025-10-13

**Authors:** Daisuke Son, Saori Nakayama, Shin-Ichi Taniguchi

**Affiliations:** 1 Community-Based Family Medicine, Faculty of Medicine, Tottori University, Yonago, JPN; 2 Administration, Institute for Community Well-Being, General Incorporated Association, Daisen-Cho, JPN

**Keywords:** book discussions, communication skills, empathy, ethical reasoning, humanities, medical education, psychological safety, qualitative research, reflective learning, social awareness

## Abstract

Background: Medical education has traditionally emphasized biomedical knowledge and technical skills; however, developing humanistic competencies, such as empathy, ethical reasoning, and communication, is equally important. Integrating the humanities and social sciences into medical curricula has been proposed as a means of fostering these competencies. Book discussion groups provide an interactive learning environment that encourages reflection, critical thinking, and engagement with diverse perspectives. Despite growing interest, limited research has examined the specific benefits of such discussions in medical education. This study investigates the impact of a book discussion group involving medical students and faculty members on participants’ perspectives, communication skills, and understanding of ethical and social issues.

Method: A qualitative study was conducted at the Tottori University Faculty of Medicine from June 2020 to May 2022. A total of 19 book discussion sessions were held, with each session attended by one to nine students. Participants were required to read an assigned book before attending a 90- to 120-minute discussion, conducted primarily online. After each session, participants completed an anonymous online survey with open-ended questions about their experiences. Thematic analysis was performed to identify key themes.

Results: Six major themes emerged from the analysis: 1) exposure to diverse perspectives, 2) deepened personal reflection and articulation of thoughts, 3) enhanced understanding of complex themes, 4) creation of a psychologically safe discussion environment, 5) increased awareness of social and ethical issues, and 6) enjoyment and intellectual stimulation. Participants reported that the discussions helped them refine their critical thinking, broaden their viewpoints, and feel more comfortable expressing their thoughts.

Conclusion: Book discussions serve as a valuable complement to traditional medical education by fostering reflection, empathy, and ethical awareness. Incorporating such initiatives into medical curricula may enhance students’ nontechnical competencies, ultimately contributing to the development of well-rounded, socially conscious physicians.

## Introduction

Medical education has traditionally emphasized the acquisition of biomedical knowledge and technical competencies essential for clinical practice. However, there is growing recognition that medical training must also foster broader humanistic skills, such as empathy, ethical reasoning, communication, and an understanding of the sociocultural contexts in which medicine is practiced [1-3]. Integrating the humanities and social sciences into medical curricula has been proposed as a means of developing these competencies, as engagement with literature, philosophy, history, and social sciences has been shown to enhance physicians’ capacity for reflection, moral reasoning, and interpersonal understanding [4-6].

One particularly effective approach to incorporating the humanities into medical education is through book discussion groups, in which students and faculty critically engage with literature that explores human experiences, ethical dilemmas, and social issues related to healthcare [7-9]. These discussions provide medical students with opportunities to engage with diverse perspectives, critically analyze narratives, and refine their ability to articulate complex ideas. Prior research suggests that structured literary discussions enhance cognitive flexibility, deepen engagement with ethical challenges, and foster a habit of critical self-reflection that extends beyond academic settings [10,11]. Furthermore, discussing literature in a collaborative setting may help develop the narrative competence essential for effective patient-centered care [12,13].

A key benefit of book discussion groups is the creation of a psychologically safe learning environment. Traditional medical education often features hierarchical structures that discourage open dialogue, making it difficult for students to express uncertainties or engage in self-reflection [14,15]. In contrast, book discussions provide a space where students can freely share their thoughts and uncertainties without fear of judgment, fostering a culture of intellectual curiosity and open communication [16,17].

Beyond personal and professional development, humanities-based discussions have been linked to improved patient care outcomes. Studies indicate that physicians who engage with literature and reflective writing exhibit higher levels of empathy, stronger communication skills, and greater awareness of social determinants of health [18-20]. By engaging with narratives that explore suffering, marginalization, and ethical conflicts, medical students can develop a more nuanced understanding of patient experiences and the broader social and structural determinants of health disparities [21-23].

Despite increasing interest in integrating the humanities into medical training, empirical research on the specific benefits of book discussion groups remains limited. While previous studies have examined the impact of narrative medicine, reflective writing, and medical humanities courses [2,24,25], fewer have explored how collaborative discussions involving students and faculty influence critical thinking, ethical reasoning, and communication skills.

Accordingly, this study aimed to examine the effects of participating in a book discussion group that engaged medical students and faculty with humanities and social sciences literature. Specifically, we addressed the following research questions: 1) How do book discussions influence medical students’ empathy, reflection, and ethical awareness? 2) In what ways do these discussions contribute to communication skills, psychological safety, and intellectual engagement in learning environments? By analyzing participants’ qualitative reflections, this study seeks to clarify how humanities-based book discussions can complement traditional medical training and foster the development of more reflective, empathetic, and socially conscious physicians.

While this study focuses on the affective and reflective domains of medical education, it complements other established educational approaches that strengthen cognitive skills and knowledge retention, together contributing to the holistic development of future physicians.

## Materials and methods

Study design

This study employed a qualitative research design to examine the effects of participation in a humanities and social sciences book discussion group among medical students and faculty members. A thematic analysis of participants' free-text responses was conducted to identify key themes related to their experiences and insights gained through the discussions.

Setting and participants

The book discussion group was organized by two faculty members from the Tottori University Faculty of Medicine and was conducted over a two-year period, from June 2020 to May 2022. A total of 19 sessions were held during this time (Table 1). Each session was attended by between one and nine medical students, primarily those enrolled in the regional quota program. Participation was voluntary, and students could join any session based on their availability and interest in the selected book. Invitations were distributed via email to all students enrolled in the regional quota program at Tottori University Faculty of Medicine. Most participants were first- to fourth-year medical students, and all identified as Japanese nationals. Most participants had limited prior exposure to formal medical humanities education, as the undergraduate curriculum at our institution includes only a brief introductory session. Therefore, for many students, the book discussions served as their first structured experience engaging with humanities perspectives in medicine. The selection of assigned books each time was determined through discussions among the faculty, aiming to choose a diverse range of books in the humanities and social sciences that would contribute to the students' learning.

**Table 1:** Summary of book discussion sessions The number of respondents represents participants who completed the post-discussion survey

Session no.	Date	Book title and author	Number of respondents	Background and positionality of the book (in relation to respondents)
1	June, 2020	The Plague; Albert Camus	9	Classic existential novel depicting epidemic and moral responsibility; resonated with students’ experiences of COVID-19 and professional duty
2	July, 2020	Paradise in the Sea of Sorrow: Our Minamata Disease; Michiko Ishimure	6	Nonfiction on environmental pollution and human suffering; prompted reflection on social justice and medical ethics in public health
3	August, 2020	The Power of Listening: An Essay on Clinical Philosophy; Kiyokazu Washida	4	Philosophical essays on empathy and dialogue; directly linked to clinical communication and listening in patient care
4	September, 2020	It’s Hard to Stay: A Book About Care and Therapy; Kaito Tohata	4	Narrative essays on caregiving and therapy; encouraged students to consider emotional labor and relational ethics
5	October, 2020	The Song of Sorrow; Shusaku Endo	3	Exploration of faith, guilt, and compassion; invited reflection on suffering and forgiveness in medical practice
6	November, 2020	The Wandering Moon; Yuu Nagira	6	Contemporary fiction addressing gender diversity and social stigma; expanded students’ awareness of inclusivity in healthcare
7	December, 2020	Writing the History of the House (Chibe); Sara Park	3	Ethnographic reflection on Korean-Japanese identity and memory; prompted discussion of cultural marginalization and narrative identity
8	March, 2021	Nausicaä of the Valley of the Wind; Hayao Miyazaki	5	Graphic novel exploring ecology and moral ambiguity; fostered reflection on coexistence and human responsibility toward life
9	April, 2021	A Life Without Borders; Mari Yamazaki	7	Autobiographical essays on intercultural experience; connected with students’ ideas about cultural humility and global medicine
10	May, 2021	Meiji, My Father, and America; Shinichi Hoshi	8	Semihistorical novel about modernization and identity; encouraged reflection on cross-cultural learning and generational values
11	June, 2021	The Alchemist; Paulo Coelho	7	Allegorical novel of self-discovery and purpose; inspired reflection on personal growth and professional calling in medicine
12	July, 2021	The Woman in the Dunes; Kōbō Abe	2	Existential allegory of entrapment and meaning; led to a discussion on perseverance and finding purpose in constrained situations
13	August, 2021	Tonan Sensei; Atsushi Nakajima	4	Story of a Confucian scholar’s moral struggle; related to ethical integrity and humility in professional life
14	October, 2021	Fahrenheit 451; Ray Bradbury	1	Dystopian critique of censorship and conformity; invited parallels with intellectual freedom in science and medicine
15	November, 2021	The Forest of Sheep and Steel; Natsu Miyashita	2	Novel about mentorship and craftsmanship; resonated with learners’ experiences of apprenticeship and skill formation
16	December, 2021	What Is 'Altruism'?; Asa Ito and Others	2	Collection of interdisciplinary essays on altruism and ethics; supported reflection on motives and moral responsibility in healthcare
17	January, 2022	Raising the Sea; Yoko Uema	1	Novel set in Okinawa addressing trauma and resilience; stimulated discussion on regional identity and postcolonial perspectives
18	April, 2022	Drive My Car; Haruki Murakami	4	Short story exploring communication and grief; connected to physician-patient empathy and emotional expression
19	May, 2022	Prison Circle; Kaori Sakagami	4	Documentary-based narrative about restorative justice; prompted reflection on rehabilitation, empathy, and social systems

Procedure

Participants were required to read the assigned book before attending the discussion session. Each session followed an open discussion format, allowing participants to share their perspectives, interpretations, and personal reflections on the book. Discussions typically lasted between 90 and 120 minutes and were conducted primarily online, except for one session (the 16th), which was held in person.

Following each discussion, participants were invited to complete an anonymous online survey with open-ended questions about their experiences. The primary question was: "What insights or realizations did you gain from the book discussion?" Participants were encouraged to express their thoughts freely without constraints.

Data analysis

A thematic analysis approach was used to analyze qualitative data from participants' responses [26]. The data were systematically coded, and recurring patterns were identified to generate overarching themes. Two researchers independently reviewed the responses to ensure reliability and consistency in theme identification. Any discrepancies in coding were resolved through discussion until consensus was reached. Coding was conducted using an inductive approach, allowing themes to emerge from the data rather than being predetermined. For example, initial codes such as “expressing personal opinions” and “hearing diverse perspectives” were later grouped into the broader theme of “exposure to diverse perspectives.” The final themes were refined through iterative team discussions to ensure consistency and analytic rigor.

Thematic saturation was reached after analyzing responses from 17 of the 19 sessions, as no new codes or themes emerged thereafter. This determination was made through iterative team discussions confirming the stability of the coding framework.

The methodological transparency of this study aligns with broader trends in medical education that emphasize reproducibility and detailed reporting. Such transparency facilitates adaptation of humanities-based learning interventions alongside other structured educational strategies designed for consistent implementation across contexts.

Ethical considerations

Participation in the study was voluntary, and informed consent was obtained from all participants. The study was conducted in accordance with ethical guidelines for research involving human subjects. To ensure confidentiality, no personally identifiable information was collected, and all responses were anonymized before analysis. The study adhered to the ethical principles outlined in the Declaration of Helsinki [27] and complied with ethical guidelines for research involving human participants. Approval for the study was obtained from the Ethics Review Committee of the Tottori University Faculty of Medicine (approval no. 21A068).

## Results

The qualitative analysis of participants' free-text responses identified several key themes regarding the effects of reading and discussing humanities and social sciences books among medical students and faculty members. The following major themes emerged.

Exposure to diverse perspectives

Participants consistently reported that engaging in discussions allowed them to encounter viewpoints different from their own, which they found highly valuable. Differences in perspectives based on gender, professional background (e.g., physicians vs. students), and life experiences were particularly noted. Some participants highlighted that the discussions broadened their understanding of how individuals interpret the same text differently: "Even when reading the same book, I was able to appreciate how people focus on different aspects and interpret them in diverse ways. It was fascinating!" "I benefited from hearing viewpoints from a variety of participants, including professionals and students, which provided fresh insights."

Deepening of personal reflections and articulation of thoughts

Many participants indicated that the discussion format encouraged deeper engagement with the texts. Knowing that they would later discuss the book prompted more careful and reflective reading. Additionally, formulating their opinions and sharing them with the group helped refine their thoughts: "Compared to reading alone, I found myself reading more attentively, knowing that I would participate in a discussion later." "I often find myself focusing on listening rather than expressing my own opinions, but I hope to gradually improve my ability to articulate my thoughts."

Enhanced understanding of complex themes

For more challenging books, participants found that discussing with others aided comprehension. Difficult concepts became more accessible through shared interpretation, and they gained new insights that they might not have arrived at independently: "Although the book was difficult, hearing others' perspectives helped me grasp its key themes more effectively." "Discussing the book from various angles-such as medical, societal, and literary perspectives-allowed me to engage with it on a deeper level."

A safe and supportive discussion environment

Over time, the book club became a psychologically safe space for participants, enabling them to share thoughts without hesitation. The presence of familiar members and a warm atmosphere contributed to this sense of security: "The discussion group felt like a small sanctuary where we could openly share our thoughts." "As I became more familiar with the members, I felt more comfortable expressing my opinions."

Increased awareness of social issues, ethics, and philosophy

Participants highlighted that reading and discussing these books helped them think critically about broader societal and ethical issues, including discrimination, injustice, and medical ethics. Some discussions also encouraged them to reflect on their own roles as future medical professionals: "Beyond deepening my understanding of Minamata disease, this discussion prompted me to consider how medical professionals should respond to such health crises." "I realized how biases and discrimination exist around us and how easily we fall into exclusionary thinking."

Enjoyment and novelty in the learning process

Participants frequently described the discussions as enjoyable and refreshing. The opportunity to explore books outside their usual reading habits was also appreciated: "I had never read this genre before, so it felt fresh and engaging." "This book club introduced me to books I wouldn't have chosen on my own, making it a rewarding experience."

The analysis reveals that reading and discussing humanities and social sciences books fosters critical thinking, enhances communication skills, and promotes deeper engagement with ethical and societal issues among medical students. Moreover, the supportive and inclusive nature of the discussions contributes to a psychologically safe learning environment. These findings suggest that such book discussions provide a valuable complement to medical education by broadening students’ perspectives and strengthening their reflective and dialogical skills.

## Discussion

This study examined the effects of a book discussion group involving medical students and faculty members reading humanities and social sciences literature. A qualitative analysis of participants' responses identified several key benefits, including exposure to diverse perspectives, deeper personal reflection and articulation of thoughts, enhanced understanding of complex themes, the creation of a psychologically safe discussion environment, increased awareness of social and ethical issues, and the enjoyment of intellectual engagement. These findings suggest that book discussions can serve as a valuable complement to traditional medical education.

One of the most significant benefits observed was the opportunity for medical students to engage with diverse perspectives. Participants consistently highlighted the value of encountering viewpoints from individuals with different backgrounds, including medical professionals, students, and faculty members. This finding aligns with previous research suggesting that exposure to diverse opinions enhances cognitive flexibility and reflective thinking in medical education [1,7]. Given that medical practice often involves navigating complex and ambiguous patient cases, the ability to consider multiple perspectives is a critical skill [7]. The book discussion format appears to foster this skill by encouraging participants to reassess their own interpretations and consider alternative viewpoints.

Effective communication is essential for medical professionals, not only in patient interactions but also within interdisciplinary teams. Our findings suggest that book discussions provide students with a platform to practice articulating their ideas clearly and engaging in meaningful dialogue. Several participants noted that they became more conscious of the need to express their thoughts coherently, a skill that is fundamental to medical practice [5]. Additionally, discussing literature encouraged students to structure their arguments logically and develop a habit of critical discourse.

Many participants reported that discussing literature deepened their understanding of social justice, medical ethics, and broader societal issues. This finding is particularly relevant given the increasing emphasis on ethical reasoning and social responsibility in contemporary medical education [6]. Books addressing themes of injustice, discrimination, and suffering, such as Paradise in the Sea of Sorrow: Our Minamata Disease and Prison Circle, prompted reflections on how societal structures impact health and well-being. These discussions may help bridge the gap between theoretical knowledge and the emotional intelligence necessary for compassionate medical practice.

Another important outcome was the creation of a psychologically safe discussion space, where students felt comfortable expressing their thoughts without fear of judgment. Psychological safety has been recognized as a key factor in effective learning environments, particularly in medical education, where hierarchical structures often discourage open dialogue [14]. The approach used in managing the book discussion group appears to have been effective in maintaining psychological safety. Participation was entirely voluntary, allowing students to join even if they had not completed the assigned reading. Furthermore, during discussions, all participants were encouraged to freely express their opinions and thoughts, ensuring an inclusive and supportive environment. The informal nature of the book discussions may have contributed to a nonhierarchical, collegial atmosphere, fostering open and honest discussions.

Participants also expressed enjoyment and appreciation for the intellectual stimulation provided by the book discussions. Many reported reading books outside their usual interests and discovering new perspectives. This finding supports the idea that exposure to the humanities can cultivate a lifelong habit of reading and inquiry, which is essential for continuous professional development in medicine [28]. Moreover, engaging with literature beyond clinical knowledge may help medical students develop greater adaptability and resilience in handling the complexities of real-world medical practice. The findings of this study contribute to the growing recognition of the role of the humanities in medical education. While traditional medical curricula primarily focus on technical and scientific knowledge, incorporating humanities-based discussions can help cultivate essential nontechnical skills, such as empathy, ethical reasoning, and reflective practice. Some medical schools have already integrated narrative medicine and medical humanities courses, and our findings further support the value of such initiatives [2]. Given the observed benefits, institutions may consider formally incorporating book discussion groups into medical training. These discussions could be structured as elective courses or extracurricular activities, allowing students to engage with literature in a guided yet flexible manner. Future research could explore the long-term impact of such initiatives on professional development, including communication skills, ethical reasoning, and patient interactions.

While this study focused on humanities-based book discussions to cultivate empathy, ethical reasoning, and critical reflection, other emerging educational strategies, such as spaced repetition, have shown promise in enhancing knowledge retention and clinical readiness. For example, Koenig et al. developed a spaced repetition model using Anki flashcards to support plastic surgery education, demonstrating improved knowledge recall and learner engagement [29]. Together, these approaches highlight the complementary roles of cognitive and affective learning strategies in shaping well-rounded physicians.

Although this study was conducted in a single regional medical school in Japan, the cultural context may shape how students perceive and engage in humanities-based discussions. Japanese educational culture often emphasizes harmony, modesty, and hierarchical respect, which may influence communication styles and willingness to express personal opinions during group discussions. Such sociocultural characteristics could both constrain and enrich reflective dialogue. Therefore, while the findings highlight the potential of book discussions in fostering empathy and reflection, their perceived value may differ in other cultural or institutional settings. Comparative studies across regions or countries could further clarify how cultural context mediates the impact of humanities-based learning.

Despite the valuable insights gained from this study, several limitations should be acknowledged. First, the findings are based on self-reported reflections, which may be influenced by social desirability bias. Additionally, participation was voluntary, and those who chose to engage in the book discussions may have already been predisposed to an interest in literature and reflective thinking. Future research employing pre- and post-discussion assessments or comparative studies with nonparticipating students could provide more objective insights into the impact of these discussions.

Further studies could also examine how book discussions influence real-world medical practice. For example, longitudinal studies tracking participants’ clinical communication skills, ethical decision-making, and patient interactions could offer stronger evidence of the educational impact of humanities-based discussions. Additionally, expanding the participant pool to include a more diverse range of students, faculty, and healthcare professionals could help generalize the findings.

## Conclusions

This study highlights the multiple benefits of a book discussion group in medical education. Engaging in discussions of humanities and social sciences literature fosters a broader perspective, enhances reflective thinking, improves communication skills, deepens understanding of ethical and social issues, and creates a psychologically safe learning environment. These discussions not only complement traditional medical training but also contribute to the development of well-rounded, empathetic, and socially conscious physicians. Incorporating such initiatives into medical curricula may help cultivate the critical thinking and ethical reasoning skills necessary for the future of medical practice.
